# Molecular profiling of thyroid cancer subtypes using large-scale text mining

**DOI:** 10.1186/1755-8794-7-S3-S3

**Published:** 2014-12-08

**Authors:** Chengkun Wu, Jean-Marc Schwartz, Georg Brabant, Goran Nenadic

**Affiliations:** 1 Faculty of Life Sciences, University of Manchester, Manchester, M13 9PT, UK; 2 Doctoral Training Centre in Integrative Systems Biology, University of Manchester, 131 Princess Street, Manchester M1 7DN, UK; 3 Manchester Institute of Biotechnology, 131 Princess Street, Manchester M1 7DN, UK; 4 Department of Endocrinology, Christie Hospital, University of Manchester, Wilmslow Road, Manchester M20 4BX, UK; 5 Experimental and Clinical Endocrinology, Med Clinic I, University of Luebeck Ratzeburger Allee 160 D-23538, Lübeck, Germany; 6 School of Computer Science, University of Manchester, Manchester M13 9PL, UK; 7 Health e-Research Centre (HeRC), Manchester M13 9PL, UK

**Keywords:** Thyroid cancer, text mining, subtype classification, molecular profiling

## Abstract

**Background:**

Thyroid cancer is the most common endocrine tumor with a steady increase in incidence. It is classified into multiple histopathological subtypes with potentially distinct molecular mechanisms. Identifying the most relevant genes and biological pathways reported in the thyroid cancer literature is vital for understanding of the disease and developing targeted therapeutics.

**Results:**

We developed a large-scale text mining system to generate a molecular profiling of thyroid cancer subtypes. The system first uses a subtype classification method for the thyroid cancer literature, which employs a scoring scheme to assign different subtypes to articles. We evaluated the classification method on a gold standard derived from the PubMed Supplementary Concept annotations, achieving a micro-average F1-score of 85.9% for primary subtypes. We then used the subtype classification results to extract genes and pathways associated with different thyroid cancer subtypes and successfully unveiled important genes and pathways, including some instances that are missing from current manually annotated databases or most recent review articles.

**Conclusions:**

Identification of key genes and pathways plays a central role in understanding the molecular biology of thyroid cancer. An integration of subtype context can allow prioritized screening for diagnostic biomarkers and novel molecular targeted therapeutics. Source code used for this study is made freely available online at https://github.com/chengkun-wu/GenesThyCan.

## Background

Thyroid cancer (TC) is the most common endocrine malignancy [1] and its incidence increase has been significant in recent years despite some controversies about the extent [2]. Many possible factors causing thyroid cancer have been reported including exposure to ionising radiation, iodine-deficiency and heredity [3]. Conventional treatment strategies include surgical resection, radiation therapy (especially radioactive iodine therapy), chemotherapy and thyroid hormone therapy [4]. However, the understanding of the underlying molecular mechanisms is still incomplete.

Thyroid tumours are usually classified into multiple subtypes according to their histopathological characteristics, and treatments are selected depending on the subtype and stage of thyroid cancer. The main subtypes include papillary thyroid cancer (PTC), follicular thyroid cancer (FTC), anaplastic thyroid cancer (ATC) and medullary thyroid cancer (MTC) [4]. PTC and FTC are also sometimes collectively referred to as differentiated thyroid cancer (DTC) or well-differentiated thyroid cancer (WDTC), while ATC can also be referred to as undifferentiated thyroid cancer. In addition, a number of rare subtypes have been described. Cellular origins and some of the known molecular mechanisms differ for each subtype [5], which may include subtype-specific alterations in DNA methylation patterns [3] and have led to new therapeutic approaches based on the molecular signature of the tumours.

These "targeted therapeutics" of thyroid cancer are being rapidly developed [6,7]. Several potential drugs are currently in preclinical testing or in clinical use [8,9]. However, lack of systematic studies of underlying molecular mechanisms can lead to a high risk for thyroid cancer patients, who might suffer from unexpected side effects. For instance, RET has been shown to be an oncogene in thyroid cancer but is considered as a potential tumour suppressor gene in colorectal cancer [10]. Consequently, studies focusing on one or a few genes are likely to miss the molecular context that could be vital for a comprehensive understanding of the disease.

**Figure 1:**
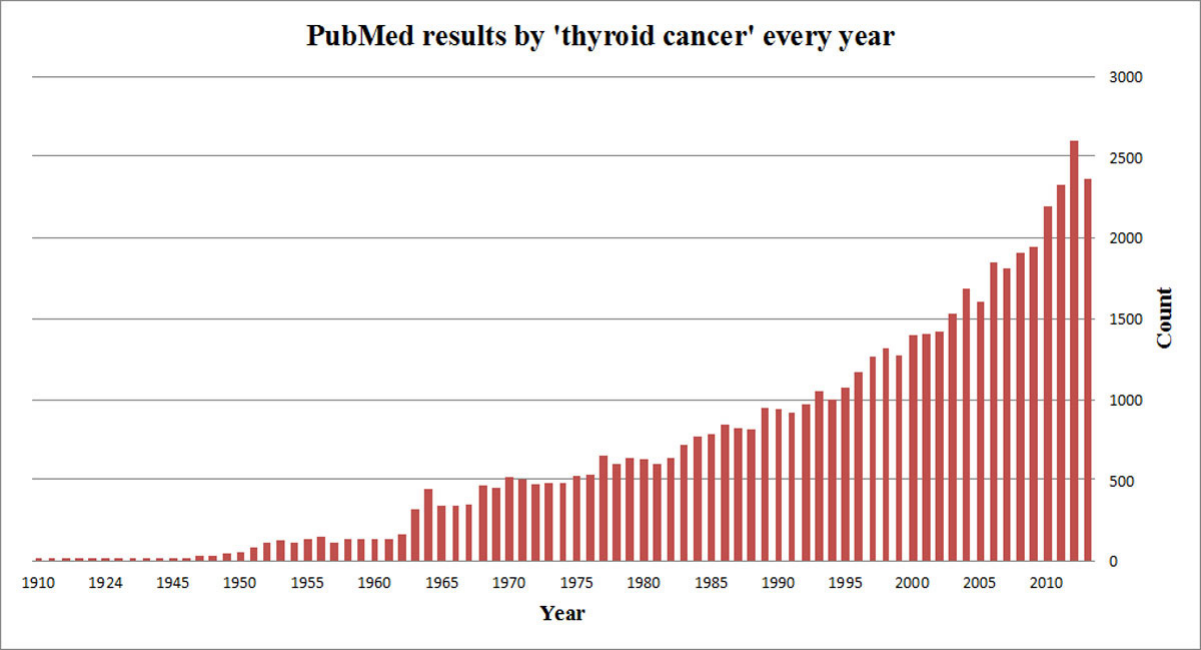
**Number of PubMed articles returned by the 'thyroid cancer' query**. The size of thyroid cancer related literature is increasing rapidly, with over 2,000 articles published annually in recent years.

For systematic studies, a major challenge is to efficiently utilise the myriad of knowledge and information from unstructured scientific literature. PubMed, one of the most widely used systems for biomedical literature search [11], returns over 50,000 results with the search query 'thyroid cancer'. The number is increasing rapidly, with over 2,000 articles published annually in recent years, as illustrated in Figure 1. This trend has made it extremely difficult for scientists to identify, retrieve and assimilate all relevant publications.

To support the identification and retrieval of relevant articles, MeSH (Medical Subject Headings, http://www.nlm.nih.gov/pubs/factsheets/mesh.html) terms are used in PubMed to label the topic of each document, along with additional headings known as Supplementary Concepts (SCs). The MeSH term used for thyroid cancer is 'thyroid neoplasms', and the major subtypes are represented as SCs. However, the thyroid cancer SCs were only introduced in August 2010, which means the majority of the thyroid cancer literature does not have a subtype label indicated by SC.

Text mining techniques have been extensively used to support access to the biomedical literature [12,13]. A number of tasks can now be successfully fulfilled by text mining, including gene mention recognition and normalization [14,15], pathway mention recognition [16] and event extraction [17,18]. Those efforts enable the extraction of structured and explicit data, and this further targets the exploration and investigation of specific biological questions.

In this paper we focus on identifying genes and pathways that are reported in the thyroid cancer literature, grouped by subtypes. We then compare our systematic effort to some established databases and demonstrate how our results could boost the understanding of thyroid cancer and promote comprehensive investigation for potential therapeutic targets.

### Related work

There are several curated databases that contain molecular information of thyroid cancer. For example, the Thyroid Cancer and Disorder Gene Database (TCGDB, http://www.juit.ac.in/attachments/tcgdb/) contains information about genes and miRNAs involved in different thyroid cancer subtypes. The data in TCGDB have been extracted through manual literature review of a few selected articles. However, details of these articles are not revealed, so it is not clear how systematic and comprehensive the database is.

MalaCards is a generic integrated compendium for diseases and associated annotations [19], with some information related to TC. It provides disease-specific information from multiple sources, including related publications, genes and pathways. However, there are several limitations: firstly, the publications associated with a disease are obtained by title search only, which could miss out many relevant articles; secondly, pathways are provided by affiliated genes rather than their mentions in the literature. Finally, the coverage of this data set is limited: for example, a search for "anaplastic thyroid cancer" returns no affiliated genes.

Gene2Pubmed [20] is a generic database maintained by NCBI that provides a set of manually added links from PubMed articles to genes. Gene2Pubmed integrates information from a number of public databases and the links are not limited to articles specifically defining the function of a given gene. However, similarly to other resources, its coverage is limited: for example, we found quite a few examples where TC-related genes were missing from the Gene2Pubmed annotation (see Results).

Several databases contain information about pathways and their associations to diseases. KEGG [21], for example, contains pathways for different diseases, but its coverage is often limited, in particular for diseases that are not widely studied (e.g. there is a single pathway diagram for thyroid cancer). Even for databases that focus on specific diseases (e.g. AlzPathway [22] is a database of pathways associated to Alzheimer's disease) it is often difficult to keep up with new findings through manual curation, and automated text mining approaches need to be used to ensure a wider coverage [16].

There are various efforts to automatically extract relationships between cancer and its molecular bases from the literature. For instance, a maximum entropy-based named entity recognizer and relation recognizer were applied to find relations between prostate cancer and genes [23]. Other relation finding tools can be applied to cancer related studies as well, including FACTA [24], CoPub [25], DigSee [26] and OncoSearch (http://oncosearch.biopathway.org). Both FACTA and CoPub support searching for co-occurring biomedical concepts (genes/diseases), facilitated by indexing through dictionary matching or regular expression matching. DigSee and OncoSearch can scan the literature to identify whether a gene is up-regulated or down-regulated in a particular type of cancer. The systems look for genes that are reported together with a biological event and cancer in the same sentence. While the results are therefore often highly accurate, they may not be comprehensive. However, no systems could support extraction of information specific to thyroid cancer subtypes.

## Methods

### Pipeline for generating the molecular profiling

**Figure 2:**
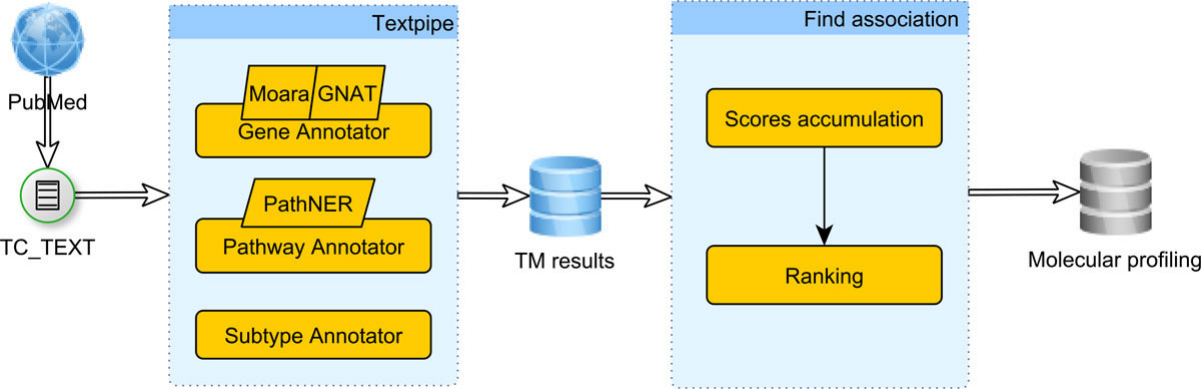
**The pipeline to generate the molecular profiling of thyroid cancer subtypes**. The pipeline starts with corpus construction, which is followed by text mining annotators implemented within the TextPipe framework. The text mining results are then used to find genes/pathways association with thyroid cancer subtypes, which lead to a comprehensive molecular profiling of primary thyroid cancer subtypes.

The pipeline we engineered to generate the molecular profiling of thyroid cancer subtypes is depicted in Figure 2. It starts with the corpus construction and is followed by the text mining tasks implemented as annotators within TextPipe [18], a framework designed for large-scale, modular text processing and data integration. Text mining results are then used to find the association between genes/pathways and thyroid cancer subtypes. These stages are explained in the following subsections.

### Data - a corpus of thyroid cancer from MEDLINE

All the documents in our corpus were retrieved from MEDLINE. We performed a PubMed search with the query "*(((thyroid neoplasms[majr] AND human[mh] AND english[la]) OR thyroid[ti]) AND (cancer OR carcinoma OR malignant OR malignancy))*", as suggested by the National Cancer Institute for retrieving the TC-relevant literature (see http://www.cancer.gov/cancertopics/litsearch/endocrine). It should be noted that the query limits the results to human studies in English. A total of 38,572 PubMed IDs (PMIDs) were fetched from PubMed (accessed on 13/01/2014). The complete list of PMIDs is available in Additional File 1. We retrieved abstracts from PubMed using those PMIDs and constructed the thyroid cancer corpus (TC_TEXT) using ***PyPubmedText***, which is freely available from https://github.com/chengkun-wu/PyPubmedText.

For the evaluation, we created the SC_GOLD corpus, which consists of all 1,213 documents with specified subtype SCs since their introduction in 2010 (see Additional File 2).

### Subtype classification using subtype scoring

For subtype classification, we aimed to label the articles in the TC_TEXT corpus with the major TC subtype(s) discussed in the associated abstract. The subtypes we investigated included PTC, ATC, FTC and MTC. We considered this as a document classification task and have developed an efficient classification method based on a subtype-scoring scheme. Although machine-learning methods are popular for document classification [27], we did not employ them here due to insufficient training data for each of the TC subtypes: while the recent articles with SCs can be used as the training data, the number of documents is still limited (1,213 in total, much fewer for each subtype). Instead, we used a flexible dictionary matching method as described below.

For each article in the corpus, we calculate a vector of scores corresponding to each TC subtype through the following steps:

1. Subtype names are often mentioned partially and in abbreviated forms, due to language economy and flexibility (e.g. "*...Inflammatory infiltrates could increase the risk of papillary cancer in patients with autoimmune lymphocytic thyroiditis*..." (PMID: 21042739)). We therefore dissociate each subtype name into three parts: (1) subtype keyword (SK); (2) anatomy keyword (AK); (3) malignancy keyword (MK). For instance, in "papillary thyroid cancer", the SK is "papillary", the AK is "thyroid" and the MK is "cancer". We then match those parts against text separately, maintaining the three separate keyword lists for matching: a subtype keyword list, an anatomy keyword list and a malignancy keyword list. For each list, possible variants are also included. For instance, "anaplastic thyroid cancer" is also named as "undifferentiated thyroid cancer", which can be written as "un-differentiated thyroid cancer". So "anaplastic", "un-differentiated" and "undifferentiated" will all be mapped to "anaplastic". The lists are included in the additional files. For matching, we applied the open-source package LINNAEUS [28].

2. Each input document is split into sentences. For each sentence, we apply dictionary matching to calculate the subtype relevance scores of that sentence calculated as follows: (1) if SK co-occurs within that sentence with both AK and MK, then the sentence has a relevance score 1 to the subtype SK; (2) if SK co-occurs with only AK or MK, then the sentence has a relevance score 0.5 to the subtype SK; (3) if SK appears alone without the presence of either AK or MK, the sentence has a relevance score 0.25 to the subtype SK. We defined these rules to cover the situations where subtype names are simplified, as illustrated above.

3. The vector of subtype scores $S_{i}=\langle S_{i}^{P},S_{i}^{A},S_{i}^{F},S_{i}^{M}\rangle$ for article $d_{i}$ is calculated by weighted accumulation of the vectors of subtype scores for each sentence (the four elements in $S_{i}$ correspond to PTC, ATC, FTC and MTC respectively). The weights of different sentences are assigned in the following way: the title of a document is considered as the most important element and is assigned a weight of 4; the first sentence in the abstract usually mentions the main topic of the document and the last sentence usually concludes the article, and both are assigned a weight of 2; the second and the penultimate sentence can be quite important as well, and are both assigned a weight of 1; other sentences in the abstract are given a weight of 0.5 in order to weaken bypassing mentions of subtype names that are not the major scope of the article.

For classification, we set threshold values for each subtype and assign the corresponding label to the article if the associated subtype score is above a pre-set value. We assigned slightly different thresholds to different subtypes, with the PTC's percentage threshold slightly higher than other subtypes, given that PTC occurs more frequently in the literature (over 50%).

### Gene recognition and normalisation

A number of tools are available for identifying mentions of genes in the literature and normalising them to database identifiers. We utilized two open source libraries, Moara [14] and GNAT [15], which have been successfully applied in other studies [18,26]. For gene name recognition, Moara utilizes the CBR-tagger [29], which treats the recognition problem as a binary classification on each token; GNAT employs dictionary-expanded regular expressions together with BANNER [30]. For the normalisation of recognized gene names, both tools map mentions to the Entrez Gene database [20], adopting similar methods for disambiguation. We wrapped Moara and GNAT as TextPipe annotators that output normalised gene mentions including document ID (PubMed ID), positions in the text, normalised Entrez Gene ID, original text and the applied tool (Moara or GNAT). If the two tools report overlapping mentions, we created a new one covering both mentions (union); if the overlapping mentions have been assigned different Entrez Gene identifiers, the priority is given to GNAT as its reported performance is higher.

### Pathway mention recognition

We utilised PathNER for pathway mention recognition from the literature [16]. PathNER is implemented using soft dictionary matching and manually created rules. For this study, we recompiled the pathway dictionary using the data from the 2013 update of the ConsensusPathDB interaction database [31]. The outputs from this annotator are biological pathway mentions including document ID (PubMed ID), positions in the text, pathway database identifier (if available) and original text.

### Association between genes/pathways and subtypes

To establish the association between text-mined information and thyroid cancer subtypes, we performed the following steps for each text-mined entity (gene or pathway, referred to as E thereafter):

1. Get all the documents $D_{E}=\left\{d_{i}|i\in \left[1,n\right]\right\}$ that mention E according to the text-mining results;

2. For each document $d_{i}\in D_{E}$, get the subtype score vector $S_{i}=\langle S_{i}^{P},S_{i}^{A},S_{i}^{F},S_{i}^{M}\rangle$ as described above;

3. Sum up all $S_{i}$ to give the subtype relevancy vector of E:

$$S_{E}=\overset{n}{\underset{i=1}{\sum }}S_{i}=\langle \overset{n}{\underset{i=1}{\sum }}S_{i}^{P},\overset{n}{\underset{i=1}{\sum }}S_{i}^{A},\overset{n}{\underset{i=1}{\sum }}S_{i}^{F},\overset{n}{\underset{i=1}{\sum }}S_{i}^{M}\rangle =\langle S_{E}^{P},S_{E}^{A},S_{E}^{F},S_{E}^{M}\rangle$$

For a given subtype j∈{P,A,F,M}, we generate a list of entities $L_{j}$, which is composed of entities that have at least one document labelled as subtype *j *in the classification stage; we then rank the entities in $L_{j}=\left[E_{1},E_{2},\cdots \;,E_{k}\right]$ in a descending order by the value of $S_{E}^{j}$ for each entity E in $L_{j}$.

## Results and discussion

### Subtype classification evaluation

For performance assessment against the gold standard, we adopted the standard metrics: *Precision (P)*, *Recall (R) *and *F1-score (F1) *defined by the following equations:

$$P=\frac{TP}{TP+FP},\;R=\frac{TP}{TP+FN},\;F1=2\frac{P\cdot R}{P+R}$$

Here, *TP *is the number of *true positives*, *FP *is the number of *false positives *and *FN *is the number of *false negatives*. Those metrics were calculated for each subtype. For documents with multiple subtype labels, the labels were evaluated separately.

The evaluation of the classification performance against the SC_GOLD corpus for each TC subtype is listed in Table 1. The performance for PTC, ATC and MTC shows good precision and moderate recall, with F1-score over 85%. This demonstrates the effectiveness of our scoring scheme.

**Table 1:** TC subtype classification performance on the SC_GOLD corpus.

		TP	TN	FN	FP	Precision	Recall	F1
**Our Method**	**PTC**	641	372	186	14	0.979	0.775	0.865
	**ATC**	114	1066	27	6	0.950	0.809	0.874
	**FTC**	21	1081	12	99	0.175	0.636	0.275
	FTC*	75	1081	12	45	0.625	0.862	0.725
	**MTC**	189	979	41	4	0.979	0.822	0.894
	**Micro Average**	1019	3498	266	69	0.937	0.793	0.859

		**TP**	**TN**	**FN**	**FP**	**Precision**	**Recall**	**F1**

**Baseline**	**PTC**	601	372	226	14	0.977	0.727	0.834
	**ATC**	109	1063	32	9	0.924	0.773	0.842
	**FTC**	19	1061	14	119	0.320	0.485	0.386
	FTC*	73	1061	14	65	0.529	0.839	0.649
	**MTC**	181	971	49	12	0.938	0.787	0.856
	**Micro Average**	964	3467	321	100	0.906	0.750	0.821

FTC* is the result after adjusting the results by considering WDTC. The micro-average calculation uses FTC*.

Unexpectedly, the precision for FTC was low (17.5%). We therefore performed error analysis to investigate the reason for that. We found that more than half of all errors (54 of the 99 FPs for FTC) are from articles about DTC or WDTC, referring to both PTC and FTC [32]. However, it seems that the PubMed SCs typically only annotate an article with PTC if it is about DTC. This is probably because the SC for FTC was introduced in August 2012, while the SC for PTC was introduced two years earlier. If those 54 cases are considered TPs then the performance is obviously improved (F1 is 72.5%; listed in Table 1 as FTC*). We also found a few other variants such as "follicular variant papillary carcinoma", "mixed medullary-follicular carcinoma of the thyroid", "follicular variant of PTC", etc. We checked the frequency of those variants by dictionary matching and we found them in 31 documents in SC_GOLD. As our classification "dissects" thyroid cancer subtype names into several parts, follicular variants will score and get incorrectly classified as FTC in those situations. The FTC precision is thus more affected as "follicular" is a word commonly used in PTC articles, since PTCs are derived from follicular cells [33].

In Table 1, we also listed the performance of a baseline method for comparison. The baseline method performs classification based on subtype name occurrences in titles and abstracts by dictionary matching using LINNAEUS [28]. The dictionary includes all available synonyms for the TC subtype names. The performance results demonstrate that our method outperforms the baseline for all subtypes.

### Application to TC_TEXT

**Figure 3:**
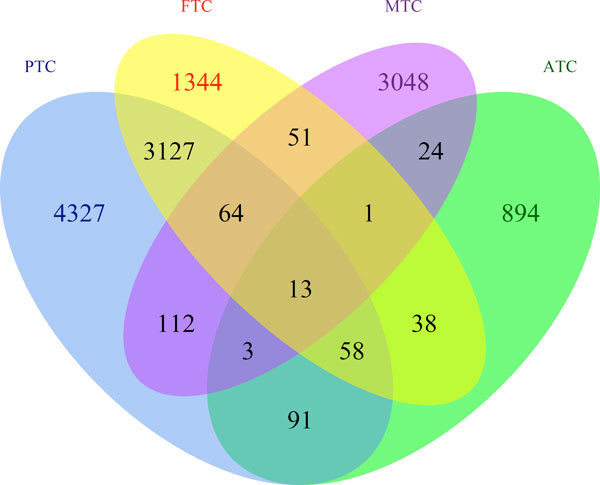
**Overlap of documents related to different TC subtypes**. The Venn diagram illustrates the overlap between documents associated to different subtypes.

We ran our classification method on the whole TC_TEXT corpus (38,572 abstracts). The complete classification results (with subtype relevancy scores and predicted labels) can be found in Additional File 3. The number of articles labelled with each subtype is listed in Table 2, which also shows the incidence rate of each subtype. We can see that the amount of literature somewhat reflects the incidence rate, with PTC as the most frequent and ATC as the least frequent among the four subtypes studied here. The results also show that it is relatively uncommon to discuss more than one subtype in an article (see Figure 3), with the exception of PTC and FTC, which are frequently studied together as they are both derived from follicular cells [33].

**Table 2:** Number of articles for each subtype in the TC_TEXT corpus.

	PTC	ATC	FTC	MTC	XTC*	TOTAL
#Articles	7795	1122	4696	3316	3768	26866
%	29.0%	4.2%	17.5%	12.3%	14.0%	
Incidence^§^	75%	1-2%	10-15%	5-10%		

XTC*: represents articles that contain more than one subtype labels.

Incidence^§ ^data source: PTC, FTC, ATC [2], MTC [39]

### Gene normalization results and gene-subtype association

**Figure 4:**
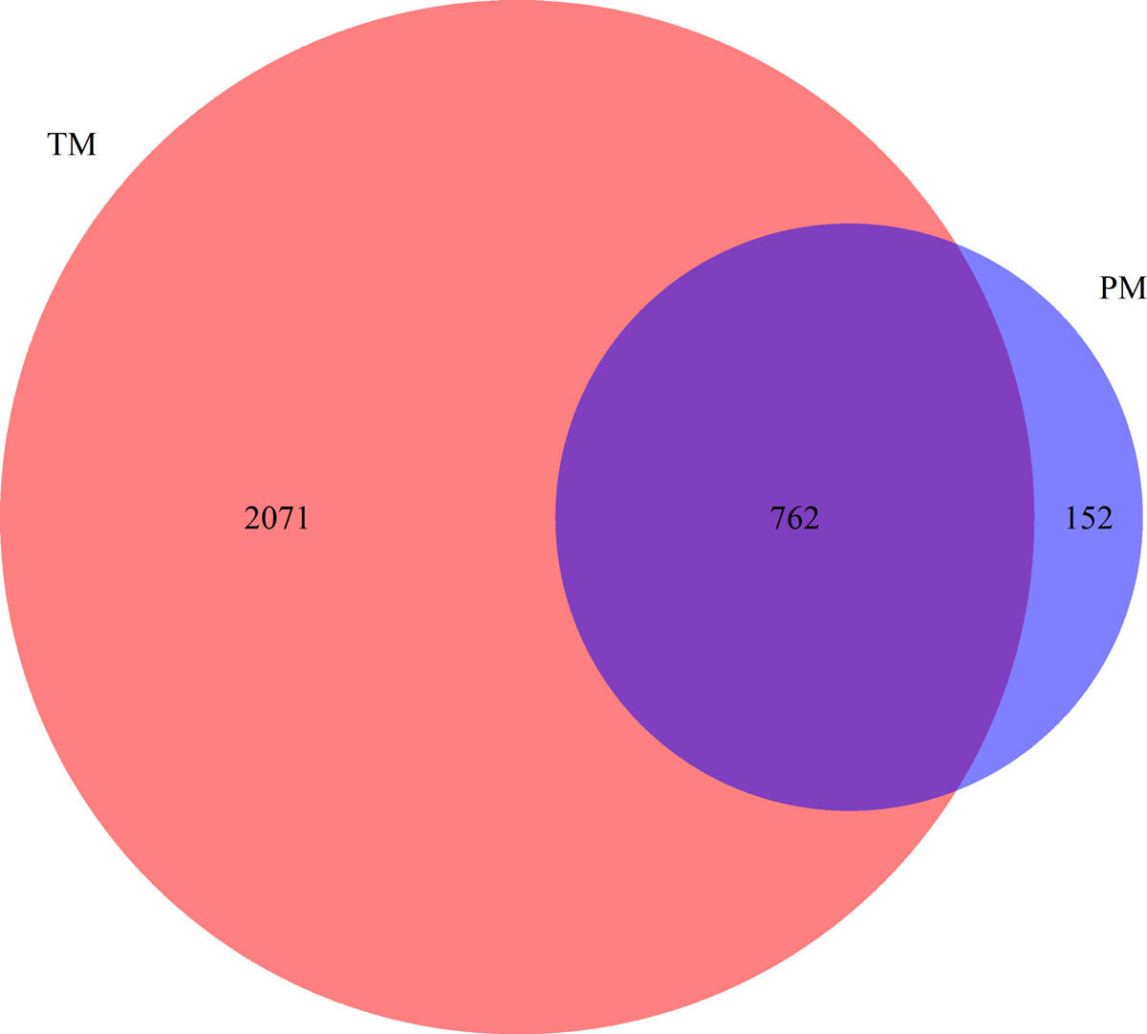
**Comparison between text-mined TC genes and Gene2PubMed**. This Venn diagram compares the text-mined genes (TM) to a manually annotated database Gene2PubMed (PM). The figure demonstrates a good coverage by text-mining and it also shows that text mining could be more comprehensive.

We ran gene recognition and normalization on the TC_TEXT corpus and a total number of 2,833 unique genes were detected. We first compared the resulting list against the gene list generated from the Gene2Pubmed database [20]. Out of 914 genes identified in Gene2Pubmed, 762 (83.4%) are found by our text mining approach. On the other hand, 2,071 out of 2,833 text-mined genes (73.1%) are not listed in the Gene2Pubmed database (see Figure 4). We randomly sampled 50 genes from that difference set and manually checked whether they are associated with TC or not. Table 3 shows these genes sorted by their document-level frequency, according to text mining results. If a gene is related to TC, an example of relevant document is given. Overall, 42 genes out of 50 are found to be reported as associated with thyroid cancer. This highlights the importance of using text mining for a comprehensive retrieval of information, complementing manually annotated data, which may potentially miss over 1,700 TC-related genes (84% of 2,071). We also note that even genes with low document-level frequencies can be relevant: for instance, *WNT7A (Entrez Gene ID: 7476) *was only detected once by text mining, but is reported to be over expressed in both PTC and its aggressive variant. Therefore, we did not use any filters based on document-level frequency in this study.

**Table 3:** Results of TC-relevance for 50 sampled text-mined genes not in Gene2PubMed.

Gene	**Freq**.	Related to TC?	PMID	Gene	**Freq**.	Related to TC?	PMID
*ENO2*	78	YES	6342746	*MUC5B*	2	NO	N/A
*SYP*	37	YES	11740050	*ADA*	2	YES	5694958
*ALB*	36	YES	591616	*MIR34A*	2	YES	24220341
*CDK2*	20	YES	23895847	*MAPK10*	2	YES	15619007
*SHBG*	12	YES	12705335	*APOD*	2	YES	14764826
*LMOD1*	9	YES	17914110	*WNT7A*	1	YES	16676402
*P4HB*	7	NO	N/A	*TSHZ3*	1	NO	N/A
*SLC2A3*	7	YES	18571834	*PMS2L11*	1	YES	21606360
*THY1*	7	NO	N/A	*FH*	1	NO	N/A
*PCSK2*	6	YES	18661512	*NQO2*	1	YES	23918565
*TNFRSF10B*	6	YES	22113498	*MAP3K5*	1	YES	20410161
*AOC1*	5	YES	191838	*HYOU1*	1	YES	20719828
*ETS1*	5	YES	11280797	*VCP*	1	YES	16189643
*AFP*	4	YES	12428567	*NDRG4*	1	NO	N/A
*CD14*	4	YES	11389034	*CLDN3*	1	YES	21606360
*STAT5A*	3	YES	21136677	*COX6A1*	1	YES	23569218
*STMN1*	3	YES	15613457	*RAPGEF3*	1	YES	11375794
*PRKAA2*	3	YES	24196587	*GNA11*	1	YES	24137342
*TAM*	3	YES	8077333	*MIR603*	1	NO	N/A
*CTLA4*	3	YES	18505566	*TAT*	1	NO	N/A
*APOE*	2	YES	17690558	*MLN*	1	YES	18844033
*HBEGF*	2	YES	23917679	*FKBP4*	1	YES	22612312
*SNAP25*	2	YES	18813355	*EPAS1*	1	YES	20578836
*TRHR*	2	YES	23781307	*MFI2*	1	YES	8090582
*YY1*	2	YES	23690926	*KLF4*	1	YES	23301671

The PMID column shows an example of document that reports evidence that a given gene is linked to TC, or N/A if we were not able to find support for the relationship.

With the subtype classification, we generated ranked gene lists for each subtype as discussed in the Methods section (see Table 4 for the statistics). The top 20 genes for each subtype are given in Table 5 (the full results are provided in Additional File 4). The genes are ranked in descending order by their subtype scores (as described in Methods section). To validate the results, we looked at the most recent review on thyroid cancer [34]. Almost all well-observed mutations (see Table 1 in [34]) are identified by our top text mining results, including *BRAF, RET*, *TP53*, *TRK (NTRK1), RAS (RASA1), PAX8-PPARG, RET/PTC (PTCH1), PTEN *and *AKT*. The only missing gene *PIK3CA *is ranked 30^th ^and 32^nd ^in the lists for FTC and ATC, respectively. This provides solid evidence for the capability of our method in assisting systematic acquisition of knowledge of the molecular biology of thyroid cancer.

**Table 4:** Number of genes extracted for each TC subtype.

	PTC	ATC	FTC	MTC	TOTAL
#Genes	1256	538	791	613	2834
%Unique	38.7%	25.5%	15.8%	40.6%	

%Unique represents the percentage of (unique) genes that appear only with a given subtype (compared to the total number of genes appearing with that subtype). So, 38.7% of genes related to PTC are unique to PTC i.e. appear only with PTC and not with other subtypes.

**Table 5:** Top 20 genes for the four thyroid cancer subtypes as extracted from the literature.

	PTC		ATC		FTC		MTC	
**Rank**	**GENE**	$S_{E}^{P}$	**GENE**	$S_{E}^{A}$	**GENE**	$S_{E}^{F}$	**GENE**	$S_{E}^{M}$

#1	TG	5471	TP53	645	TG	4246	CALCA	6173
#2	RET	4916	TG	424	TSHB	1039	RET	3880
#3	BRAF	2582	AKT1	274	RET	585	SST	1016
#4	PTCH1	1829	CDKN1A	231	PAX8	453	TG	547
#5	TSHB	1175	EGFR	228	TSHR	435	CEACAM3	462
#6	NCOA4	1115	CASP3	213	BRAF	419	GAST	367
#7	RASA1	914	BRAF	209	RASA1	392	CEACAM19	365
#8	TP53	819	RET	179	PPARG	374	CHGA	353
#9	LGALS3	693	PAX8	151	LGALS3	364	POMC	334
#10	KRT19	662	RASA1	151	TP53	333	TXK	325
#11	MAPK1	658	BCL2	144	CALCA	272	ENO2	260
#12	TSHR	570	CCND1	141	TPO	266	NTRK1	224
#13	CCDC6	557	VIM	137	PPBP	235	RASA1	209
#14	NTRK1	422	VEGFA	121	AKT1	222	TP53	206
#15	HBE1	386	NKX2-1	120	KRT19	193	EGFR	177
#16	NKX2-1	384	CALCA	119	PTEN	174	ELL	177
#17	AKT1	371	PARP1	111	EGF	173	AKT1	169
#18	VEGFA	369	TXK	111	PPARA	167	KDR	162
#19	PPBP	343	PPARG	108	NKX2-1	156	SSTR1	161
#20	MET	329	SLC5A5	107	EGFR	150	BCL2	147

We also compared the subtype gene lists generated by text mining to the manually created TCGDB database (see Table 6): only a small number of genes for each subtype were missed by our method. The majority of the genes in the subtype lists from TCGDB have been successfully retrieved by our method, along with a number of candidates for further manual curation.

**Table 6:** Text-mined genes compared with the TCGDB database.

	PTC	ATC	FTC	MTC
#TCGDB	60	15	10	20
#TM Missed	8	2	3	2
%Coverage	86.7%	86.7%	70%	90%

The first row represents the total numbers of genes in TCGDB associated to a given subtype. The second row represents the number of genes missed by text mining. The final row is the coverage: (#TCGDB-#TM Missed) / #TCGDB.

**Figure 5:**
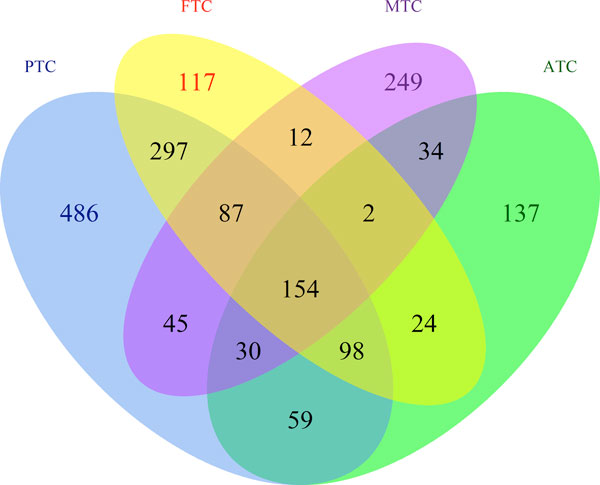
**Overlap of genes associated to different TC subtypes**. This figure shows a Venn diagram of gene lists for the four subtypes and their overlap.

Figure 5 shows a Venn diagram of gene lists shared between the four subtypes. Compared to Figure 3, we observe a much higher degree of overlap among subtypes. This indicates that a considerable number of common genes are investigated across different subtypes but in separate studies. Meanwhile, each subtype is still characterized by a significant number of unique genes.

### Pathway mention recognition results

Table 7 presents the top 20 pathways for each TC subtype (For the complete text mining results for TC related pathways, please refer to Additional File 5). The most frequently mentioned pathways are typical biological processes involved in most cancers, including apoptosis, angiogenesis, cell cycle, etc.

**Table 7:** Top 20 pathways for the four thyroid cancer subtypes as extracted from the literature.

Top	PTC		ATC		FTC		MTC	
	
	Pathway	Evidence	Pathway	Evidence	Pathway	Evidence	Pathway	Evidence
#1	Apoptosis	22120515	Apoptosis	20067110	Apoptosis	24213562	Apoptosis	10614665
#2	Cell cycle	23231932	Cell cycle	22688732	Angiogenesis	14605010	Cell cycle	21973234
#3	MAPK pathway	23544999	Angiogenesis	17575107	Cell cycle	19190121	Angiogenesis	20133461
#4	Angiogenesis	23528368	PI3K/Akt pathway	22918703	PI3K/Akt pathway	18492751	RET pathway	15316058
#5	DNA Repair	21860547	MAPK pathway	17989125	DNA Repair	22331172	mTOR pathway	22136849
#6	S Phase	22329804	S Phase	18813835	S Phase	2874658	S Phase	18791128
#7	PI3K/Akt pathway	22744707	M Phase	22399519	MAPK pathway	18492751	Focal Adhesion	12850460
#8	Glucose transport	21606885	NF-kB pathway	19158360	PI3K pathway	23128507	MAPK pathway	15746253
#9	PI3K pathway	20804548	Glucose transport	12667615	Focal Adhesion	20225271	Hedgehog (Hh) pathway	23410206
#10	Wnt/beta-catenin pathway	23261982	Focal Adhesion	19293266	Oxidative Stress	22331172	Notch1 pathway	18520232
#11	TGF-beta pathway	21874046	Wnt pathway	15650354	TGF-beta pathway	10942134	bone remodeling	6611007
#12	mTOR pathway	21822208	Glycolysis	3155492	Glucose transport	16273245	mRNA Processing	2582437
#13	Oxidative Stress	9774495	Notch1 pathway	23594881	thyroid hormone production	N/A	Raf-1 pathway	17363508
#14	MAPK/ERK pathway	22426956	G1 Phase	9038381	Glucose metabolism	19433487	PI3K pathway	17188151
#15	MEK/ERK pathway	20629553	mTOR pathway	20689131	cAMP pathway	N/A	Notch pathway	20182588
#16	cAMP pathway	21479404	Hedgehog (Hh) pathway	23860623	thyroid hormone biosynthesis	N/A	ERK activation	21470995
#17	Focal Adhesion	22513979	STAT3 pathway	22328572	Cytokinesis	15886755	PI3K/Akt pathway	23934677
#18	Notch pathway	23544172	Wnt/beta-catenin pathway	17218945	Hedgehog (Hh) pathway	N/A	Glucose transport	9426419
#19	Glycolysis	23846818	p21 pathway	22918703	Glycolysis	N/A	EGFR pathway	22025146
#20	Glucose metabolism	20473281	Rb/E2F pathway	15118916	VEGF pathway	18509004	Glycolysis	3155492

"Evidence" column presents one example PMID that reports a given relationship (N/A: Not Applicable if there is no evidence)

To validate these results, we compiled a list of pathways from the most recent review on the molecular biology of thyroid cancers mentioned above [34]. The list includes five pathways: the mitogen-activated protein kinase (MAPK) pathway, the phosphatidylinositol 3-kinase (PI3K)/Akt signaling pathway, the Wnt/beta-catenin pathway, the NF-kB pathway and the Hypoxia-Inducible Factor (HIF)-1α pathway. Except for the HIF-1α pathway, all other pathways are present in the top 20 pathways extracted for subtypes by text mining (see Table 8). The HIF-1α pathway was reported to be a potential therapeutic target for thyroid cancer [35] and has been experimentally verified in a couple of cell lines [36]. However, this pathway is absent from the text-mined results, even though the reporting paper [36] was included in TC_TEXT corpus. Our method did detect the HIF-1α pathway mention and found its association with thyroid carcinomas. However, the subtype relevancy scores for that abstract (PMID: 19808899) are low. The reason is that the cell lines used in the study were actually subtype-specific but this information was only available in the full-text. This example suggests the importance and potential of the analysis of full text articles as opposed to abstracts, which is an objective for future work.

**Table 8:** Reviewed TC related pathways and text-mined evidence for each subtype.

Pathway	Related to TC?	Evidence PMIDs			
		
		PTC	ATC	FTC	MTC
MAPK pathway	YES	16896265	16410725	21196179	23934677
PI3K/Akt signaling pathway	YES	18000091	22918703	17426084	23329180
Wnt/beta-catenin pathway	YES	22204713	17218945	18727708	N/A
NF-kB pathway	YES	23528368	19885592	16314832	N/A
HIF-1α pathway	YES	N/A	N/A	N/A	N/A

For other pathways in Table 7 that have not been mentioned in the above review, we have further investigated whether they are associated with the corresponding subtypes. For instance, the Notch signalling appears in the top 20 pathways for PTC, ATC and MTC but was not mentioned in [34]. We however found direct literature evidence that links this pathway to TC: the Notch pathway has crosstalk with the MAPK pathway and affects the PTC proliferation [37], and the activation of the Notch signalling has been identified as the potential therapeutic strategy for ATC [38]. We followed this procedure for each pathway in Table 7: an example PMID is given in the "Evidence" column, which represents an article that reports the association between the subtype and the pathway (if applicable). Almost all pathways in the top 20 list are supported by literature evidence: only for five pathways in the FTC list we were not able to find support in the literature. This demonstrates the potential of our work for the systematic and comprehensive acquisition of knowledge of thyroid cancer.

## Conclusions

In this paper we presented an approach to finding molecular information associated with different subtypes of thyroid cancer. We developed a method for subtype classification and performed text mining to identify genes and biological pathways associated with each subtype. The generated gene and pathway lists provide a comprehensive compendium of the key molecular information related to thyroid cancer subtypes.

The data and results from our study form the basis for a further comprehensive analysis of the molecular biology of thyroid cancer, enriched by subtypes. Based on the molecular differences between subtypes revealed in our study, biologists can look for better diagnostic biomarkers. In addition, the molecular information can be used to build molecular networks of thyroid cancer subtypes, which would enable further systems biology analyses and stimulate the development of targeted therapeutics.

## Availability

The code and data files are available at https://github.com/chengkun-wu/GenesThyCan.

## List of abbreviations

NER: Named Entity Recognition; PMID: PubMed ID; TC: Thyroid Cancer; PTC: Papillary Thyroid Cancer; FTC: Follicular Thyroid Cancer; ATC: Anaplastic Thyroid Cancer; MTC: Medullary Thyroid Cancer; DTC: Differentiated Thyroid Cancer; WDTC: Well-Differentiated Thyroid Cancer; MeSH: Medical Subject Headings; SC: Supplementary Concept; TCGDB: Thyroid Cancer and Disorder Gene Database; SK: Subtype Keyword; AK: Anatomy Keyword; MK: Malignancy Keyword; TP: True Positives; FN: False Negatives; FP: False Positives;

## Competing interests

The authors declare that they have no competing interests.

## Authors' contributions

CW developed the system for the detection on the subtype-genes/pathways association, and drafted the manuscript. GN provided support and guidance from the text mining perspective and JMS from the systems biology perspective. GB provided expertise on thyroid cancer. GN and JMS conceived and supervised the project. CW, JMS, GB and GN wrote the manuscript. All authors read and approved the final manuscript.

## Supplementary Material

Additional File 1 **TC_TEXT PMID list**. The PMIDs in the TC_TEXT corpus are listed in this .txt file, one PMID per line. Click here for file

Additional File 2 **SC_GOLD PMID list**. The .txt file is the gold standard derived from the MeSH Supplementary Concepts for thyroid cancer. It consists of 1,213 PMIDs and their corresponding thyroid cancer SCs. The values in the file are tab separated. Click here for file

Additional File 3 **Classification results with scores and subtype labels**. The .xlsx (Excel) file contains the classification results on the TC_TEXT corpus with subtype relevancy scores and predicated subtype labels. If a document is not related to TC at all, it is given the label "NON"; if a document is related to TC but without a specific focus on one or multiple subtypes, it is given the label "TC"; if a document is assigned multiple subtype labels, the labels are separated by '|'. Click here for file

Additional File 4 **Text-mined gene results**. The .xlsx (Excel) file contains the text mining results for TC-related genes. The file contains a mapping between TC-related documents and their associated genes, categorized by subtypes. Click here for file

Additional File 5 **Text-mined pathway results**. The .xlsx (Excel) file contains the text mining results for TC-related pathways. The file contains a mapping between TC-related documents and their associated pathways, categorized by subtypes. Click here for file

## Declarations

Publication of this article was funded by the UK Biotechnology and Biological Sciences Research Council (BBSRC) through the Doctoral Training Centre for Integrative Systems Biology at the University of Manchester.

This article has been published as part of *BMC Medical Genomics *Volume 7 Supplement 3, 2014: Thirteenth International Conference on Bioinformatics (InCoB2014): Medical Genomics. The full contents of the supplement are available online at http://www.biomedcentral.com/bmcmedgenomics/supplements/7/S3.
